# *Toxoplasma gondii* infection as a risk factor for osteoporosis: a case–control study

**DOI:** 10.1186/s13071-022-05257-z

**Published:** 2022-04-27

**Authors:** Kehui Zhu, Kun Liu, Junsi Huang, Xueqiong Weng, Qiaoyun Chen, Tianyu Gao, Kebing Chen, Chunxia Jing, Jing Wang, Guang Yang

**Affiliations:** 1 Department of Pathogen Biology, School of Medicine, Jinan University, Guangzhou, 510632 Guangdong China; 2 Institute of Blood Transfusion, Chinese Academy of Medical Sciences and Peking Union Medical College, Chengdu, 610000 Sichuan China; 3 Department of Epidemiology, School of Medicine, Jinan University, No.601 Huangpu Road West, Guangzhou, 510632 Guangdong China; 4 Department of Orthopedics, The First Affiliated Hospital of Jinan University, Guangzhou, 510630 China; 5 Department of Spine Surgery, Center for Orthopaedic Surgery, The Sixth Affiliated Hospital of Sun Yat-Sen University, Guangzhou, 510655 China

**Keywords:** *T. gondii*, Osteoporosis, Infection, IgG

## Abstract

**Background:**

More than one-third of the total world population is infected by *Toxoplasma gondii* (*T. gondii*). *T. gondii* has been linked to various diseases, such as cancer, mental disorders, type 2 diabetes mellitus (T2DM), etc. However, the effects of *T. gondii* infection on the risk of osteoporosis are unclear. Our study aimed to uncover evidence to determine whether patients exposed to *T. gondii* have an increased or decreased risk of osteoporosis in people with abnormal bone mineral density (BMD) by using case–control study.

**Methods:**

A total of 729 patients, including 316 osteopenia and 413 osteoporosis patients of Han Chinese ancestry were selected in the study. Their blood samples were collected and the levels of specific IgG antibodies against *T. gondii* were measured using ELISA assay. We obtained some information about the patients from the medical record that included demographic indexes and clinical data. A logistic regression analysis was used to evaluate the effects of *T. gondii* infection on femur osteoporosis, lumbar osteoporosis and compound osteoporosis. Potential interaction was analyzed using multifactor dimensionality reduction software 1.0.0 (MDR 1.0.0).

**Results:**

113 positive patients with *T. gondii* infections have been detected, including 80 cases of osteoporosis and 33 cases of osteopenia, the infection rates of *T. gondii* were 19.37% (80/413) and 10.44% (33/316), respectively. The patients with *T.gondii* infections were at a 2.60 times higher risk of suffering from compound osteoporosis than those without *T. gondii* infections (OR = 2.60, 95% CI 1.54–4.39, *P* < 0.001), but not associated with femur osteoporosis (OR = 1.01, 95% CI 0.43–2.34, *P* = 0.989) and lumbar osteoporosis (OR = 0.84, 95% CI 0.34–2.07, *P* = 0.705) after adjusting for the covariates. Moreover, a significantly higher risk of compound osteoporosis in the individuals with all two factors (*T. gondii* infection, Female) was observed compared with reference group (without *T. gondii* infection, male) under the interaction model (OR = 11.44, 95%CI = 5.44–24.05, *P* < 0.001). And the individuals with all two factors (*T. gondii* infection, over 70 years) exhibited a 8.14-fold higher possibility of developing compound osteoporosis compared with reference group (without *T. gondii* infection, under 70 years) (OR = 8.14, 95% CI 3.91–16.93, *P* < 0.001). We further stratified by age and sex, and found that women with *T. gondii* infection was more likely to develop compound osteoporosis than those without infection(OR = 3.12, 95% CI  1.67–5.81, *P* < 0.001), but we not found the association between *T. gondii* infection and compound osteoporosis in males (OR = 1.36, 95% CI 0.37–4.94, *P* = 0.645).

**Conclusions:**

*T. gondii* infection is a risk factor for osteoporosis, especially compound osteoporosis. Meanwhile, it is very necessary for patients with osteoporosis to further diagnose and treat *T. gondii* infection, especially women.

**Graphical Abstract:**

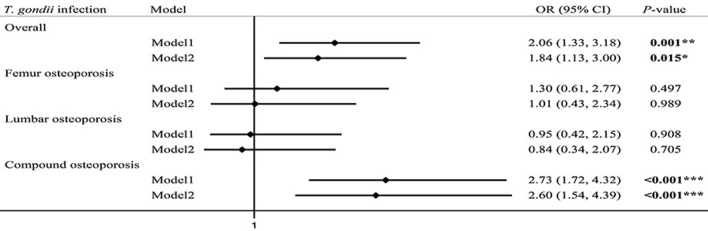

**Supplementary Information:**

The online version contains supplementary material available at 10.1186/s13071-022-05257-z.

## Background

Osteoporosis, characterized by the degeneration the microstructure of bone tissue and the decrease of bone mass, has become a public health problem affecting more than 200 million people worldwide [1, 2]. Clinically, osteoporosis will increase the fragility and susceptibility to fracture and osteoporosis-related fractures are common causes of morbidity and death in older adults [2–4]. Among the Chinese population aged 50 years or older, 65 million people are diagnosed with osteoporosis, while an additional 213 million were estimated to have osteopenia [5]. Zhang et al. reported that the prevalence of osteoporosis in Chinese women after menopause reach up more than 60% [6]. It brings great health and economic burden to old women [7].

Osteopenia refers to the bone mineral density (BMD) lower than the normal population but higher than the bone density of the osteoporotic population. Although the people with osteopenia had an increased fracture risk compared with the healthy, it was not severe enough to be considered a diseased state in the absence of a fragility fracture, if left untreated, they can eventually lead to osteoporosis and future fractures [6, 8, 9]. Most osteoporosis are asymptomatic, which makes epidemiological research especially difficult [10].

Gender, age, history of fractures, drinking, smoking, bone diseases and lack of physical activity are well-known risk factors for osteoporosis, particularly calcium deficiency, inadequate Vitamin D intake, and deficiencies in certain hormones, such as estrogen [11–14]. Furthermore, genetic polymorphisms also play a role in the development of osteoporosis [15]. Interestingly, it has been found that schistosome infection promotes osteoclast-mediated bone loss in mice [16]. However, the relationship between *Toxoplasma gondii* infection and osteoporosis is still unclear.

*Toxoplasma gondii* (*T. gondii*) belongs to apical complex protozoa, an important opportunistic pathogenic protozoan that infects almost all endotherms, including mammals, birds, and humans [17]. *T. gondii* can replicate and invade almost any nucleated cell in humans [18–20]. As an obligate intracellular parasite, It forms vacuoles in cells during infection, parasitizes in vacuoles and secretes effector molecules to regulate host cell biological processes such as energy metabolism, immune response, cell signaling, and lead to cell lysis to death along with the reproduction of *T. gondii* [21]. About 2 billion people worldwide are chronically infected with *T. gondii*, affecting approximately 30–50% of the world's population [22–24]. In a total of 49,784 Chinese blood donors from 1986 to 2017, the infection rate of *T. gondii* was detected at 6.26% [25]. Clinically, most infections are asymptomatic or taken in a mild, self-limiting form characterized by fever, malaise and lymphadenopathy [26]. *T. gondii* can lead to serious illnesses and even death of immunodeficiency patients [27]. Primary infection in pregnant women is a matter of great concern, the women in the first and second trimesters, infection with *T. gondii* may cause severe congenital toxoplasmosis, and can result in intrauterine fetal death and spontaneous abortion [28].

*T. gondii* has been linked to a various diseases.*T. gondii* infection is a serious problem in cancer patients in a case–control study [29]. And some studies have found that *T. gondii* might be a factor associated with hypertension in type 2 diabetes mellitus (T2DM) patients [30]. Moreover, *T. gondii* has been linked to a variety of mental disorders, such as schizophrenia, Alzheimer diseases, obsessive–compulsive disorder, recurrent migraines and even suicidal behavior [31–35]. Furthermore, *T. gondii* infection leads to deficits in goal-directed behavior in healthy elderly by altering dopaminergic neural transmission [36]. However, the chronic long-term damage to human health caused by latent *T. gondii* infection is not entirely clear, especially osteoporosis.

Until now, there is a lack of research supporting the relationship between *T. gondii* infections and osteoporosis in human. Therefore, our study aimed to uncover evidence to determine whether patients exposed to *T. gondii* have an increased or decreased risk of osteoporosis in people with abnormal bone mineral density (BMD) by using case–control study.

## Method

### Patients

A total of 729 osteopenia and osteoporosis patients of Chinese ancestry were included in our study, there were 316 osteopenia and 413 osteoporosis cases. All of these were inpatients at the Guangzhou Overseas Chinese Hospital from 2015 to 2019. And 5 ml peripheral venous blood sample from each patient was collected with the EDTA vacuum blood collection tubes and saved at 4℃, then transported to laboratory. Our study was approved by the Ethics Committee of the School of Medicine of Jinan University, Guangzhou, China, and performed strictly in accordance with the Declaration of Helsinki.

### Data collection

We obtained some information about the patients from the medical record that included demographic indexes (age, gender, marriage, education, job, smoking, drinking), clinical data including, bone mineral density (BMD) of whole-body, lumbar and femur, diabetes, hypertension and cardiovascular disease (CVD), and current osteoporosis treatment. This information and dual-energy densitometry (DXA) report were led together by date.

### The diagnosis of osteopenia and osteoporosis

Clinically, osteoporosis can be screened by physical examination. However, BMD measured by DXA is needed to confirm such a diagnosis, DXA is the gold standard for diagnosing osteoporosis [37]. Before DXA inspection, patients were instructed to rest at least 8 h during the previous night and to avoid strenuous exercise and alcohol consumption for 24 h, and during the measurement, subjects were in light clothing. The radiologist asked the patient to lie flat on the machine bed, the legs were fully extended, and the lower extremities were internally rotated (45 degrees), and if necessary, the lower limbs were fixed to expose the femoral neck as much as possible. When testing the whole body and lumbar spine, the patient just needs to lie down. In general, the entire inspection is maintained for 15 to 20 min.

The BMD value of an individual patient is expressed in terms of the number of standard deviations (SD) from the mean BMD of a healthy young-adult reference population, commonly referred to as the T-score [38]. Patients whose DXA showed low BMD by World Health Organization (WHO) guidelines were identified: individuals were considered to be osteoporosis when the T-score was below − 2.5 (T-score ≤ − 2.5) and were considered to be normal when the T-score was above − 1.0 (T-score > − 1.0), the BMD value of osteopenia is less than − 1.0 but above − 2.5 (T-score ≤ − 1.0 and > − 2.5) [39–43].

### Serological analysis

Approximately 5 ml of venous blood was drawn from each patient and then centrifuged at 1000 ×*g* for 10 min. Serums were separated from the blood sample and stored at – 80 ℃. Seroprevalence of *T. gondii* infection was assessed by enzyme-linked immunosorbent assay (ELISA) Kit by Haitai Biological Pharmaceuticals (registration number: 20153400072). Positive, negative serum controls, and three critical control were included in each plate. The results (A value) were read by a microplate reader (TZCAN-SAFIRZ-Z) and Magellan software at the dual wavelength of 450/630 nm. Follow the manufacturer instructions, each experiment needed to fulfill the following three conditions: (1) the mean value of A in the positive control was ≥ 0.50; (2) the mean value of A in the negative control was ≤ 0.10; (3) the A values in the critical control range from 0.12 to 0.35. When A value of the sample is greater than the average value of the critical control group, it is judged as positive, otherwise negative.

### Statistical analysis

Statistical analysis was performed using the statistical software SPSS (version 13.0). For the continuous variables were compared using Student’s *t* test. For categorical variables, the Chi-square test was used to determine associations between osteoporosis and potential risk factors, the strength of the associations was assessed by odds ratios (OR) and 95% confidence intervals (CI) were calculated. The continuous variables were reported as the means ± standard deviation. The frequency and proportion were reported for the categorical variables. Multivariate logistic regression models were used to adjust for potential confounders. Additionally, potential interaction was analyzed using multifactor dimensionality reduction software 1.0.0 (MDR 1.0.0). Results were considered significant at *P* < 0.05.

## Results

### Demographic characteristics

A total of 729 patients with abnormal BMD of Chinese ancestry were included in our study, 56.65% with osteopenia and 43.35% with osteoporosis. The mean ages of people with osteopenia, and osteoporosis were 67.28 ± 9.64 years, and 71.89 ± 9.60 years, and there was significant difference in age (*t* = − 6.41, *df* = 727, *P* < 0.001). The proportion of female in the osteoporosis group (83.05%) was higher than that in the osteopenia group (65.51%) (χ^2^ = 29.75, *df* = 1, *P* < 0.001). The proportion of high triglyceride (TG > 1.5 mmol/L) in the osteopenia group (42.46%) was higher than that in the osteoporosis group (31.69%) (χ^2^ = 8.22, *df* = 1, *P* = 0.004). In this study, 113 cases of *T. gondii* infections have been detected, and the *T. gondii* infection rates of osteoporosis and osteopenia were 19.37% and 10.44%, respectively, and there was significant difference (χ^2^ = 10.89, *df* = 1, *P* = 0.001). No differences were observed between the groups in smoking, drinking, hormone taking, job, total cholesterol level (TC), and the number of comorbidities including hypertension, diabetes and cardiovascular disease (CVD) (Table 1).

**Table 1:** Characteristics among the population with abnormal BMD (% within group)

Variables	Overall (*N* = 729)	Osteopenia (*N* = 316)	Osteoporosis (*N* = 413)	*P*-value
Age/years (Mean ± SD)	69.89 ± 9.88	67.28 ± 9.64	71.89 ± 9.60	< 0.001***
Sex				
Male	179 (24.55)	109 (34.49)	70 (16.95)	< 0.001***
Female	550 (75.45)	207 (65.51)	343 (83.05)	
Job				
Retire	292 (40.05)	126 (39.87)	166 (40.19)	0.995
Worker/farmer	117 (16.05)	52 (16.46)	65 (15.74)	
Others	274 (37.59)	118 (37.34)	156 (37.77)	
Missing	46 (6.31)	20 (6.33)	26 (6.30)	
Smoking				
No	715 (98.08)	310 (98.10)	405 (98.06)	0.970
Yes	14 (1.92)	6 (1.90)	8 (1.94)	
Drinking				
No	699 (95.88)	298 (94.30)	401 (97.09)	0.060
Yes	30 (4.12)	18 (5.70)	12 (2.91)	
Hormone taking				
No	684 (93.83)	294 (93.04)	390 (94.43)	0.439
Yes	45 (6.17)	22 (6.96)	23 (5.57)	
TG				
≤ 1.5 mmol/L	427 (63.73)	164 (57.54)	263 (68.31)	0.004**
> 1.5 mmol/L	243 (36.27)	121 (42.46)	122 (31.69)	
TC				
≤ 4.5 mmol/L	243 (36.27)	98 (34.39)	145 (37.66)	0.383
> 4.5 mmol/L	427 (63.73)	187 (65.61)	240 (62.34)	
Number of comorbidities				
0	426 (58.44)	172 (54.43)	254 (61.50)	0.135
1	195 (26.75)	95 (30.06)	100 (24.21)	
≥ 2	108 (14.81)	49 (15.51)	59 (14.29)	
*T. gondii* infection				
No	616 (84.50)	283 (89.56)	333 (80.63)	0.001**
Yes	113 (15.50)	33 (10.44)	80 (19.37)	

*BMD* bone mineral density, *TG* triglyceride, *TC* total cholesterol, *CVD* comorbidities including hypertension, diabetes and cardiovascular disease, *SD* standard deviation

^*^P < 0.05; **P < 0.01; ***P < 0.001;

### Risk of osteoporosis associated with *T. gondii* infection

**Fig. 1:**
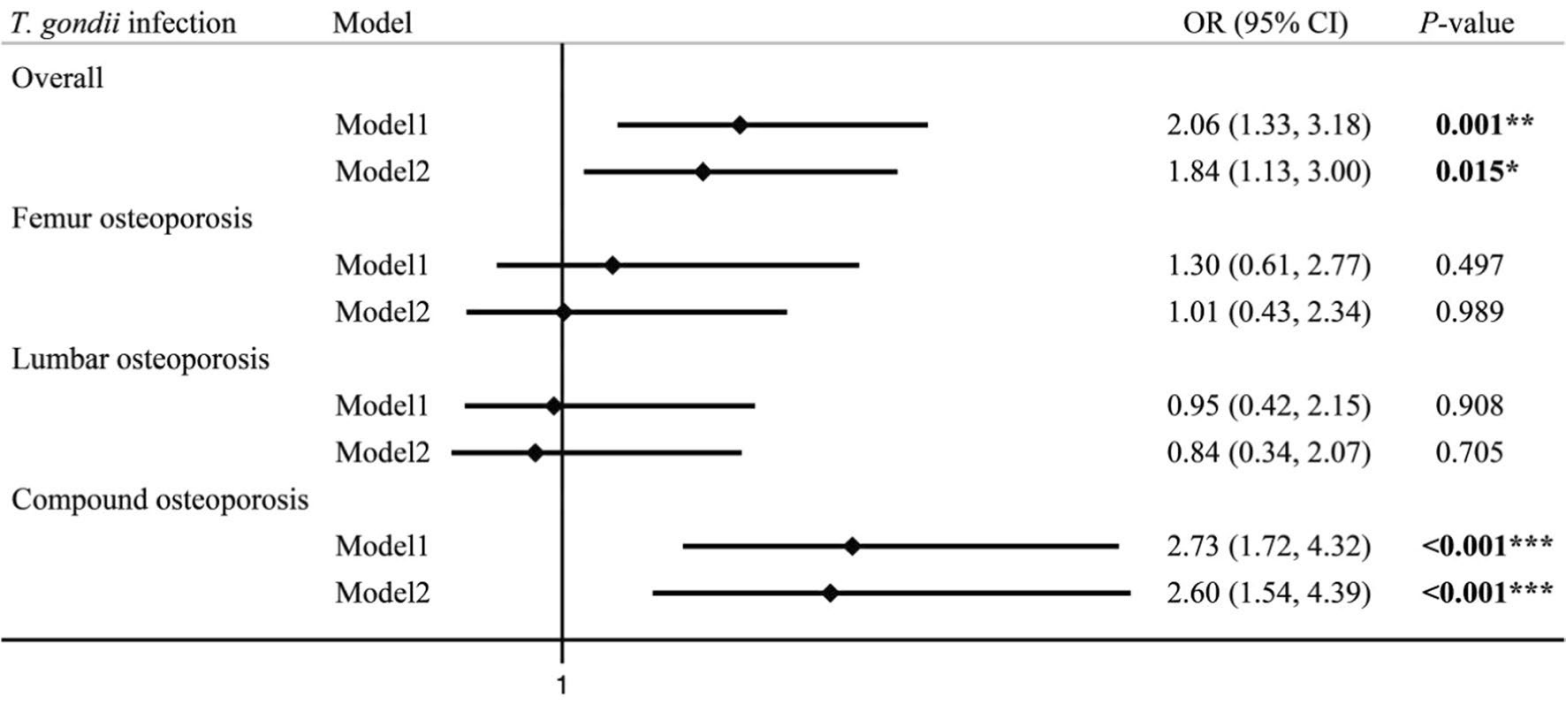
Logistic regression model of the associations between three different types of osteoporosis and *T. gondii* infection. Note: *P < 0.05, **P < 0.01, ***P < 0.001, Model 1: unadjusted model; Model 2: adjusted for age, sex, job, smoking, drinking, hormone, TG, TC, number of comorbiditie

Among the 413 patients with osteoporosis, according to the different sites of osteoporosis, we divided them into three groups: femur osteoporosis, lumbar osteoporosis and compound osteoporosis (including both femoral and lumbar osteoporosis or whole-body osteoporosis), and the osteopenia was regarded as the control group. A logistic regression analysis showed that patients with *T.gondii* infections were at a 2.60 times higher risk of suffering from compound osteoporosis than those without *T. gondii* infections (OR = 2.60, 95% CI 1.54–4.39, *P* < 0.001) (Table 2) after adjusting age, sex, job, smoking, drinking, hormone taking, TG, TC, number of comorbidities. There were no statistically significant differences among the femur osteoporosis and lumbar osteoporosis (Fig. 1).

**Table 2:** Risk of osteoporosis associated with *T. gondii* infection

outcome	*N* (%)	Mode1			Mode2		
		OR	95%CI	*P*-value	OR	95%CI	*P*-value
femur osteoporosis	76 (18.40)	1.30	0.61–2.77	0.497	1.01	0.43–2.34	0.989
lumbar osteoporosis	80 (19.37)	0.95	0.42–2.15	0.908	0.84	0.34–2.07	0.705
Compound osteoporosis	257 (62.22)	2.73	1.72–4.32	< 0.001***	2.60	1.54–4.39	< 0.001***

Model 1: unadjusted model

Model 2: adjusted for age, sex, job, smoking, drinking, hormone taking, TG, TC, number of comorbidities

*P < 0.05, **P < 0.01, ***P < 0.001

### The interaction models of compound osteoporosis

We summarized the best interaction models for different types of osteoporosis by MDR, the best model was determined by the testing balanced accuracy (TBA) and cross-validation consistency (CVC) indices. *P* values obtained from the MDR analysis among three groups. Our results revealed that age and sex have an interactive effect on compound osteoporosis (*P* = 0.039) (Table 3).

**Table 3:** Best MDR interaction models for osteoporosis

outcome	Best models	Interaction testing balanced accuracy (%)	CVC	*P*
Femur osteoporosis	Job, TG	58.42	10/10	0.402
Lumbar osteoporosis	Sex, job, Number of comorbidities	57.03	8/10	0.476
Compound osteoporosis	Sex, Age	63.11	10/10	0.039*

*CVC* cross validation consistency, *TG* triglyceride

^*^P < 0.05; **P < 0.01; ***P < 0.001

We further analyzed the interactive effects of *T. gondii* infection with age or sex on compound osteoporosis. In comparison with the reference group (without *T. gondii* infection, male), the individuals with all two factors (*T. gondii* infection, Female) exhibited a 11.44-fold higher possibility of developing compound osteoporosis (OR = 11.44, 95% CI 5.44–24.05, *P* < 0.001) (Table 4). In the interaction of *T. gondii* infection and age, the individuals with all two factors (*T. gondii* infection, over 70 years) were 8.14 times more likely to suffer from compound osteoporosis when compared with reference group (without *T. gondii* infection, under 70 years) (OR = 8.14, 95% CI 3.91–16.93, *P* < 0.001) (Table 5). We further stratified the population by age and sex, and found that women with *T. gondii* infection was more likely to develop compound osteoporosis than those without infection (OR = 3.12, 95% CI 1.67–5.81, *P* < 0.001), but we not found the association between *T. gondii* infection and compound osteoporosis in males (OR = 1.36, 95% CI  0.37–4.94, *P* = 0.645) (Additional file 1: Table S1). Furthermore, *T. gondii* infection was associated with compound osteoporosis in women under 70 years (OR = 4.35, 95%CI = 1.79–10.57, *P* = 0.001) and over 70 years (OR = 2.48, 95% CI 1.03–6.01, *P* = 0.044) (Additional file 2: Table S2).

**Table 4:** Different interaction models (with sex) for compound osteoporosis

*T. gondii* infection	Female	Overall	OR	95% CI	*P*-value
−	−	123	1	–	–
+	−	22	1.57	0.51–4.83	0.429
−	+	355	3.82	2.24–6.54	< 0.001***
+	+	73	11.44	5.44–24.05	< 0.001***

adjusted for age, job, smoking, drinking, hormone, TG, TC, number of comorbidities

^*^P < 0.05; **P < 0.01; ***P < 0.001

**Table 5:** Different interaction models (with age) for compound osteoporosis

*T. gondii* infection	Age(≥ 70)	Overall	OR	95% CI	*P*-value
−	–	248	1	–	–
+	–	39	3.74	1.70–8.25	0.001**
−	+	230	4.29	2.71–6.78	< 0.001***
+	+	56	8.14	3.91–16.93	< 0.001***

Adjusted for sex, job, smoking, drinking, hormone, TG, TC, number of comorbidities

^*^P < 0.05; **P < 0.01; ***P < 0.001

## Discussion

In order to find out whether *T. gondii* infection is related to the occurrence of osteoporosis in patients with abnormal BMD, we collected a total of 729 blood samples with osteoporosis and osteopenia between 2015 and 2019, and collected corresponding demographic and clinical information. The IgG antibody against *T. gondii* was measured by ELISA, and 113 (113/729, 15.50%) cases of *T. gondii* infection were found, is obviously higher than the average level in China (10%) [44]. In this study, women account for a much larger proportion in osteoporosis (83.05%) than the group of osteopenia (65.51%). This is consistent with the results of other studies that women are more likely to suffer from osteoporosis, especially postmenopausal women [45, 46]. This is because estrogen deficiency in postmenopausal women leads to reduce bone mass by approximately 10%, and it can be as high as 20% in those 5–6 years around menopause [47]. We found that the osteopenia group had more people with high TG (> 1.5 mmol/L) and our results was consistent with Dennison et al. research in which a significant positive correlation between fasting TG levels and lumbar spine BMD by cohort study was observed [48].

Osteopenia is the disease progression process of osteoporosis, in other words, osteopenia is a necessary condition for osteoporosis [49]. Interestingly, we found that patients with osteoporosis have a higher proportion of *T. gondii* infection (19.37%) than osteopenia group (10.44%). *T. gondii* can infect all nucleated cells (including bone marrow cells), in animal studies, Brazilian *T. gondii* Laboratory (LabTXOP) extracts high concentrations of *T. gondii* DNA from the bones sample (vertebrae and ribs) of mice [50], and some studies have shown that *T. gondii* may present serious effects on bone marrow cells in human bone marrow transplantation [51, 52]. At the cellular level, osteoblast and osteoclast are two main types of cells that maintain bone mass [53]. In normal circumstances, osteoblasts and osteoclasts maintain a certain number and constrain each other, the bone formation and bone resorption mediated by them are in balance [14]. However, the decreased bone marrow cells and decreased osteogenesis are important factors leading to osteoporosis, and it has been proved that chronic inflammatory response or inflammation caused by acute infection will increase the activity of osteoclasts, osteocytes are decomposed and absorbed, and eventually lead to the loss of bone mass[54]. And many inflammatory mediators have been implicated in driving osteoclast-mediated bone destruction [55, 56]. Indeed, TNF is a potent osteoclastogenic agent [57]. IL-12, TNF-α and IFN-γ are important cytokines produced after *T. gondii* infection [58]. Therefore, we propose that *T. gondii* infection may be a risk factor for osteoporosis. The possible mechanism is that the immune response to *T. gondii* infection activates osteoclasts, resulting in bone resorption over bone formation, which needs to be proved by further experiments.

In order to further analyze the type of bone that *T. gondii* affects osteoporosis, we divided osteoporosis into three different types of osteoporosis (femur, lumbar and compound), logistic regression showed that *T. gondii* infections were at a 2.60 times higher risk of compound osteoporosis than those without *T. gondii* infections (OR = 2.60, 95% CI 1.54–4.39, *P* < 0.001), but there is no significant difference from other types of osteoporosis (femur and lumbar). Obviously, *T. gondii* infection is a risk factor for osteoporosis, especially compound osteoporosis. On the one hand, the BMD in this study was measured by iDAX, and the precision of the BMD of the compound (0.7%) is higher than lumbar and femur (0.8%) measured by iDAX [59]. This may make compound osteoporosis caused by *T. gondii* easier to detected. On the other hand, compound osteoporosis includes osteoporosis of lumbar, femur, skull, ribs, etc. This may be that *T. gondii* is more likely to affect more active, rich blood vessels, and more vulnerable bone parts, such as skull, ribs, etc. But it needs to be confirmed by more detailed epidemiological data.

Some studies have shown that age and gender have an interactive effect on bone microstructure [60]. We also considered whether the *T. gondii* infection has an interaction effect with age and sex on compound osteoporosis, and found that women infected with *T. gondii* and people over 70 years infected with *T. gondii* have a higher risk of compound osteoporosis than other people. The results of the stratified analysis suggest that *T. gondii* infection needs to be monitored in women to prevent compound osteoporosis.

Our research has a potential limitation. Worldwide genotypic analysis of *T. gondii* isolates has identified a population structure consisting of three widespread clonal lineages, termed type I, II, and III [61], and each displays are distinct biological traits, such as virulence. In the existing article shows that the genotype Chinese 1 (ToxoDB#9) type II strain is very popular in southern China [62]. Therefore, the type II strain may be the main type affecting whole-body bone mineral density values in patients with osteoporosis. Selection bias from one hospital cannot be avoided completely and this is one limitation. In the future research, a large sample size from multiple hospitals is needed to confirm the relationship between *T. gondii* infection and osteoporosis risk. In addition, this was a hospital-based case–control study and we want to explore the relationship between *T. gondii* infection and osteoporosis progression. Since the blood samples are collected in the orthopedics department in the hospital, it is difficult for us to obtain blood sample with normal BMD. Obviously, using osteopenia as a control group would greatly underestimate the risk of *T. gondii* on osteoporosis. Although the use of osteopenia as a control might underestimate the risk, the results will provide the important clues for the future research. Next we will conduct community-based case–control study using a health control. In this study, we found positive association between *T. gondii* infection and osteoporosis..

In conclusion, our study shows that *T. gondii* infection is a risk factor for osteoporosis, especially compound osteoporosis. Meanwhile, it is very necessary for patients with osteoporosis to further diagnose and treat *T. gondii* infection, especially women.

## Supplementary Information

**Additional file 1:** **Table S1.** Risk of *T. gondii* for compound osteoporosis under different stratification factors.

**Additional file 2:** **Table S2.** Risk of *T. gondii* for compound osteoporosis in women stratified by age.

## Data Availability

Not applicable.

## Abbreviations

TG: Triglyceride
TC: Total cholesterol
SD: Standard deviation
BMD: Bone mineral density
*T. gondii*: *Toxoplasma* *gondii*
MDR: Multifactor dimensionality reduction
CVC: Cross-validation consistency
