# Supplementary material for: Toxoplasma gondii infection as a risk factor for osteoporosis: a case–control study
Source: Parasit Vectors. 2022 Apr 27;15:151. doi: 10.1186/s13071-022-05257-z (PMC9044867; doi:10.1186/s13071-022-05257-z)
Supplement: Supplementary file 1 — Additional file 1: Table S1. Risk of T. gondii for compound osteoporosis under different stratification factors. [file 13071_2022_5257_MOESM1_ESM.docx]

**Table S1** Risk of *T. gondii* for compound osteoporosis under different stratification factors

| Variable | OR (95% CI) | *P*-value |
| --- | --- | --- |
| Age group (years) |  |  |
| <70 | 3.93 (1.77-8.75) | **0.001**** |
| ≥70 | 2.05 (1.01-4.17) | **0.049*** |
| Sex |  |  |
| Male | 1.36 (0.37-4.94) | 0.645 |
| Female | 3.12 (1.67-5.81) | **<0.001***** |

Note: *, *P*<0.05; **, *P*<0.01; ***, *P*<0.001;

adjusted for age, sex, job, smoking, drinking, hormone, TG, TC, number of comorbidities with exception of stratifying factors.
