# Supplementary material for: Toxoplasma gondii infection as a risk factor for osteoporosis: a case–control study
Source: Parasit Vectors. 2022 Apr 27;15:151. doi: 10.1186/s13071-022-05257-z (PMC9044867; doi:10.1186/s13071-022-05257-z)
Supplement: Supplementary file 2 — Additional file 2: Table S2. Risk of T. gondii for compound osteoporosis in women stratified by age. [file 13071_2022_5257_MOESM2_ESM.docx]

**Table S2** Risk of *T. gondii* for compound osteoporosis in women stratified by age

| Variable | OR (95% CI) | *P*-value |
| --- | --- | --- |
| Age group (years) |  |  |
| <70 | 4.35 (1.79-10.57) | **0.001**** |
| ≥70 | 2.48 (1.03-6.01) | **0.044*** |

Note: *, *P*<0.05; **, *P*<0.01; ***, *P*<0.001;

adjusted for job, smoking, drinking, hormone, TG, TC, number of comorbidities with exception of stratifying factors.
